# Creation of a Custom Endoluminal Vacuum-Assisted Device for Salvage of Ileal Pouch-Anal Anastomotic Leak

**DOI:** 10.7759/cureus.49754

**Published:** 2023-11-30

**Authors:** Isabel Kiko, David Nehring, Truong Ma

**Affiliations:** 1 General Surgery, Summa Health, Akron, USA; 2 Surgery, Summa Health, Akron, USA

**Keywords:** colorectal, negative pressure woundtherapy, negative pressure, anastomotic leak, endoluminal intervention, trans-anal drainage, ileal pouch-anal anastomosis, endoluminal vacuum, colorectal anastomotic leak

## Abstract

Endoluminal vacuum-assisted devices have become increasingly popular solutions for low-colorectal injuries and anastomotic defects in recent years. We present a case of a 23-year-old male who underwent salvage of an ileal pouch-anal anastomosis (IPAA) anastomotic leak with a customized endoluminal vacuum sponge and cavity marsupialization. This case highlights the easy-to-follow steps to create a customized endoluminal vacuum (endo-vac) sponge with readily available materials for the treatment of low colorectal anastomotic leaks. Included are step-by-step photos and instructions for successful endoluminal vacuum device construction.

## Introduction

According to Guyton et al., the reported rates of anastomotic leak following ileal pouch-anal anastomosis (IPAA) vary widely from 5% to 19%, with the majority of this contributing data coming from single institution case series [1]. Following an uncontrolled leak discovery, the goals of treatment are patient resuscitation and infection source control. Gaining abscess source control is traditionally accomplished through draining procedures, either operatively or through radiologic guidance. Draining is the hallmark treatment for removing the nidus for infection, gathering cultures for antibiotic guidance, and promoting cellular healing in the region [2].

More recently, endoluminal vacuum-assisted devices have instead been utilized to treat colorectal leaks in non-peritonitic, clinically stable patients [2,3]. A Shalaby et al. meta-analysis reports a success rate of 85.3% for partial or complete healing of the anastomotic defect with 75.9% stoma reversal in 267 patients using endoluminal vacuum-assisted therapy [4].

Endoluminal vacuum-assisted device kits themselves can be costly or, even more likely, difficult to locate if not well-stocked at various institutions. In this case, we present successful patient treatment of IPAA leaks with wound vac materials that are relatively low-cost, easy to use, and readily available.

## Case presentation

**Figure 1:**
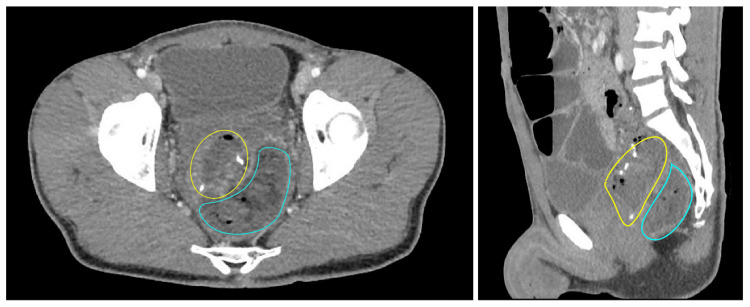
Axial and sagittal views of IPAA with staple lines outlined in yellow with posterior abscess/leak cavity outlined in blue on CT abdomen pelvis with contrast.

This is a case of a 23-year-old male with a delayed diagnosis of Hirschsprung disease who was taken for robotic-assisted laparoscopic completion proctectomy and mucosectomy with hand-sewn IPAA with diverting loop ileostomy. The patient had a previous total colectomy for the megarectum and megacolon after years of constipation. The patient tolerated the procedure well but experienced prolonged postoperative ileus and posterior pelvic abscesses. The patient had a percutaneous drain placed into the pelvic abscess found near the ileo-anal anastomosis by radiologic guidance. The patient tolerated the drain well and was subsequently discharged. At follow-up, the patient was noted to have continued purulent fluid in the drain and similar purulent discharge from the anus. The patient was admitted for a rectal examination under anesthesia (REUA) to evaluate anastomosis and was found to have a small anterior dehiscence with granulation tissue and a posterior anastomotic leak with a large abscess cavity, as depicted in Figure 1.

The goal of therapy in this young, relatively healthy patient with diverting loop ileostomy was the salvage of the anastomosis, and therefore, the following steps were taken. The anterior defect was closed primarily with 2-0 Vicryl stitches. For the posterior defect, a custom endoluminal vacuum sponge was created and placed into the defect. Three days later, the patient returned to the operating room for a REUA and a vacuum sponge change. Two days after this, he returned to the operating room, and it was found that the patient’s defect cavity had decreased in size with visible granulation tissue and decreased purulence. It was decided at this time to marsupialize the cavity and incorporate it into the J-pouch. The division of the septum between the posterior sinus cavity and pouch body was made with three white loads of an Endo GIA 60 mm stapler. The patient tolerated the procedure well and was subsequently discharged. At follow-up, the patient is continuing to heal, and plans are for later diverting ostomy reversal.

## Discussion

**Figure 2:**
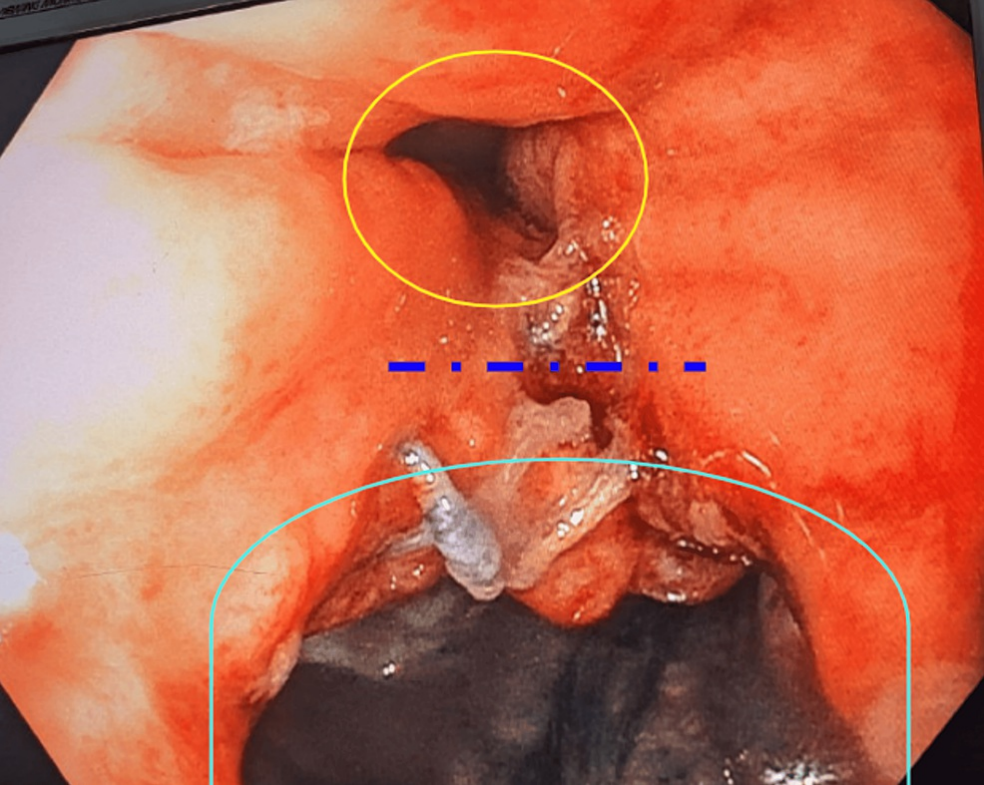
True lumen outlined in yellow and abscess cavity outlined in cyan with dashed indigo line representing septum as marked on image, seen on initial evaluation of abscess cavity prior to endoluminal vacuum placement.

**Figure 3:**
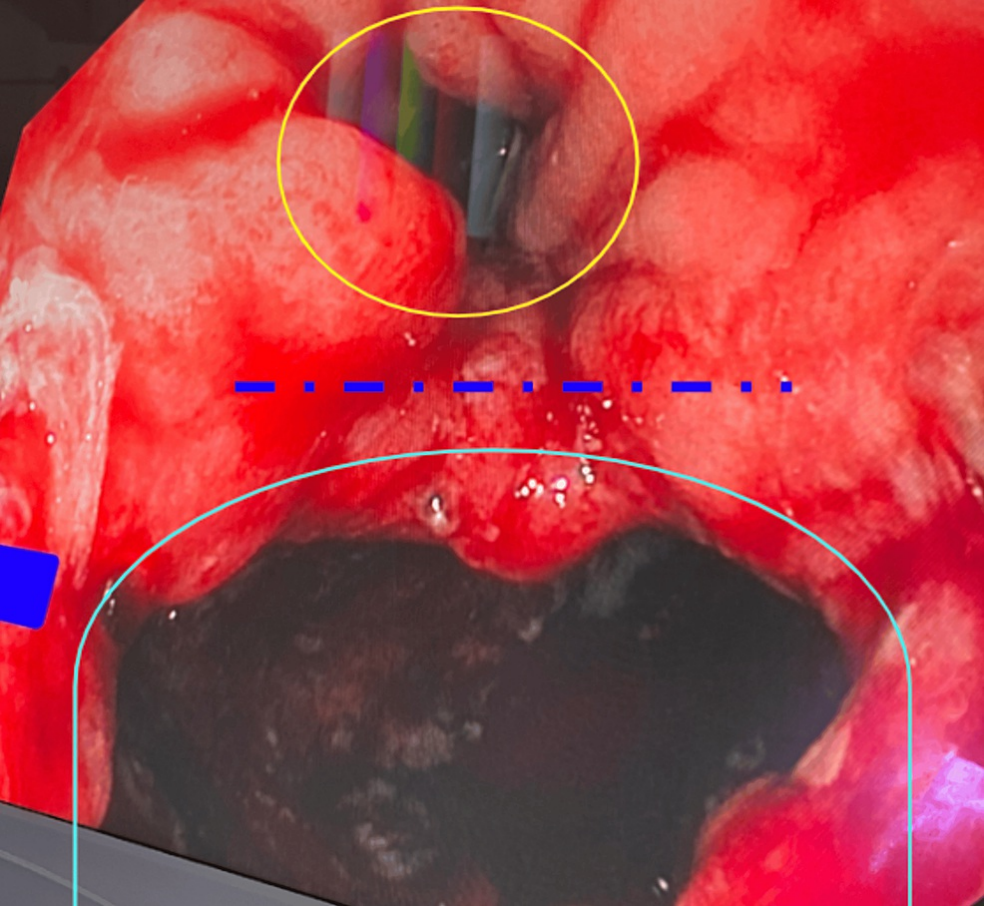
True lumen outlined in yellow and posterior abscess cavity outlined in cyan with dashed indigo line representing septum as marked on image, seen with granulation tissue and decreased purulence after just a few days of vacuum therapy.

**Figure 4:**
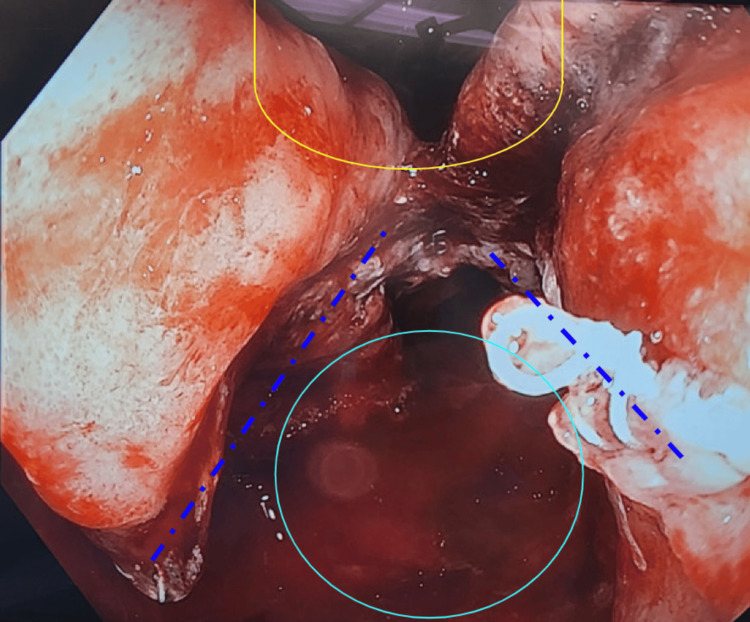
Final resulting pouch after marsupialization of defect cavity into J-pouch; true lumen outlined in yellow and posterior defect cavity opening marked in cyan with divided septum and staple lines marked with indigo dashed lines above.

The benefits of negative-pressure wound therapy have been well documented and avidly utilized in complicated surface wounds. Figures 2-4 below exhibit the progression of healing and tissue granulation facilitated by endoluminal vacuum therapy. This method of treatment facilitated the source control necessary to salvage the anastomosis.

The benefit of creating our own vacuum sponge is that it is customizable to the size and shape of the abscess cavity. In addition to this, the endoluminal vacuum is just as well tolerated by patients compared to traditional trans-anal drainage with malecot tubes but has greater suctioning ability to control pelvic sepsis. Percutaneous drains are fraught with problems of dislodgement and clogging, which can hinder their ability to control infection. The endo-vac materials are also cost-effective and readily available. Specifically marketed endoluminal vacuum sponges, more commonly utilized in esophageal injury management, can be expensive and may not be accessible at every institution. These methods show an avenue for treatment that is effective and reproducible in similar patients.

Drawbacks to the use of vac-assisted treatment for low colorectal and coloanal anastomotic leaks are the lack of long-term data and the lack of indications or clear clinical guidelines for usage [2]. Conglomerates of data on the subject show wide variability in success rates. One such meta-analysis of 20 articles by Sharp et al. quotes the successful treatment of anastomotic leak with endoluminal negative pressure therapy being between 60% and 100% [5]. Results are promising, but further research is still warranted for measuring success and longevity.

**Figure 5:**
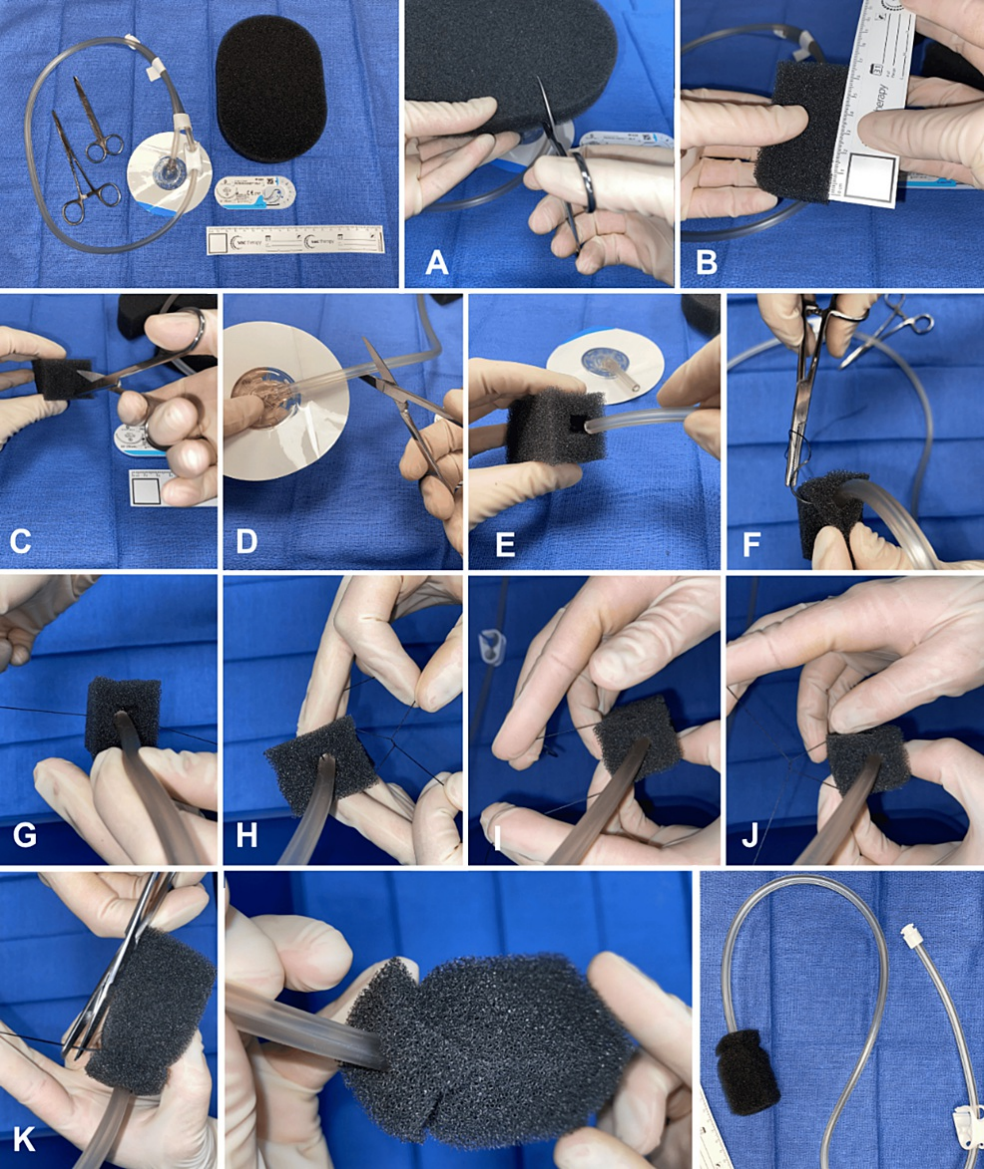
Steps for creation of vacuum sponge in alphabetical order (A-K), with top left photo as starting materials and bottom right two photos as final product.

The following are the steps taken to create the endo-vac device: first, the cavity was interrogated and measured. A medium-black GRANUFOAM sponge was cut to the dimensions of the cavity. Using scissors, a rectangular notch was cut into one end of the sponge for the insertion of the tubing. The lily pad was then cut from the vacuum tubing. Using a 0 silk suture, the sponge was attached to the vacuum tubing placed inside the rectangular notch. The stitch was placed through the sponge and vacuum tubing and was secured tightly with surgical knots on two opposing sides. A small amount of lube was placed on the end of the sponge, and it was inserted into the abscess cavity. Once inserted into the defect, another 0 silk suture was used to secure the distal tubing to the buttock to prevent dislodgement of the sponge. The tubing was placed under continuous 125-mmHg suction. Steps 1-11, for visual steps on how to create the device, are shown in Figure 5.

## Conclusions

Overall, negative pressure endoluminal therapy can help promote healing and trans-anal drainage in anastomotic leaks. Endoluminal vacuum-assisted therapy with custom sponge materials is an effective and reproducible treatment in our patient’s case with readily available hospital wound vac resources. Continued research on the subject is warranted, but so far, outcomes are promising.
